# Cundurango

**Published:** 1871-10

**Authors:** 


### Cundurango—How to Treat Humbugs.

Probably there is no class of men, unless it be the clergy, who have the credit of being more dogmatic and intolerant than physicians, and yet there is probably no class who could, on demand, show a better reason for the faith that is in them. This is doubtless due to the fact that the medical man has to deal with the practical application of a science which it is impossible for him to explain to the uninitiated, within the ordinary limits of the intercourse existing between a physician and patient or patient's family. It is necessary, therefore, that he should possess their confidence, and that they should be content to confide to him the treatment of the patient, without expecting to play the doctor themselves, or to have explained to them in a moment what it takes ordinary intellects years to master. We have no patience with those old women in the profession who consult with every old woman out of the profession and spend half their lives in a vain effort to preach and expound bad therapeutics and worse pathology to the understanding of their befogged but flattered patrons.

There is, however, a field where it appears to us that the medical profession might, with propriety and advantage, give the public more information and satisfaction than it does, thereby doing much to relieve itself from the charge of blind intolerance, and at the same time rendering the community a valuable service.

Let physicians first thoroughly inform themselves, if need be, and then truly and quietly inform the people, as they have occasion, why certain patent medicines are worthless or dangerous, or entirely inapplicable to nine-tenths of the diseases they claim to cure; how certain pretended oculists, aurists, electricians, etc., never had an opportunity to study the specialties they claim to practice; what are the errors and absurdities of certain so called schools of medicine, how impossible it is for their followers to practice according to the principles they profess to believe, and how, if they do not do this, they are not honest men. These are some of the foes that we have constantly in the field, while individual skirmishers and blockade runners of every description are ever appearing on the horizon, worthy perchance of a random shot if not of a more protracted chase.

What is the custom of physicians to-day when questioned by their patients or friends on any of these subjects? Is it not most brusquely, contemptuously, and with scant courtesy even toward the questioner, to burst forth with "fraud!" "quack!" "humbug!" giving no reason for the sentence thus summarily passed, and even growing indignant if asked for a reason? The imposture seems to them so plain and aggravated that they can scarcely have patience with any one for questioning it. And yet no doubt this very method of treating the subject has led many of our friends into the belief that there is something in these frauds, that they are dangerous competitors of the doctor, and that therefore he waxes so indignant when they are mentioned. If the physician had calmly explained the matter to them, and given the data for his opinion, he might, nine times out of ten, have convinced them of the inefficacy of a preparation or the incapacity of a man in which or in whom they have now invested not only faith but money.

In this connection we take pleasure in calling the attention of our readers to a valuable article in the *Chicago Medical Examiner*, for September, 1871, on "Homœopathy as it was and is," Dr. G

W. Earle. This paper gives us chapter and verse for a good many of the absurdities of Hahnemannism, sufficient, one would suppose, to convince any candid mind that the system has nothing to entitle it to the confidence of reasonable people.

Physicians throughout the country are to-day being asked by the more intelligent reading portion of the community, "What of Cundurango? Is it a cure for cancer?" Merely and curtly to answer, "no, it's a humbug," will have the effect of making many a man doubt the soundness of our judgment or the extent of our information on the subject. Let us then, from the start, meet this new fraud, for such we consider it to be, with facts and not merely with sneers.

Specimens of this drug were distributed in April last by the Department of State to the Surgeon General of the Army, the Chief Medical Officer of the Navy, the head of the Department of Agriculture, and some others, for the purpose of having its merits tested. It has been subjected to a chemical analysis by Prof. Antisell, of Washington, D.C., and Dr. E. R. Squibb, of Brooklyn, N.Y., both of them gentlemen of national reputation. They find it to contain no new active principle of any kind, rank it, therapeutically, among the aromatic bitters, and expect nothing more from it, as the result of analysis, than from any other variety of the same class.

In practice, the drug has been tried by medical officers of the army and navy, residing in Washington, New York, and elsewhere, and having the best opportunity for selecting cases of cancer seated in various organs and at different stages of progress. In every case *Cundurango has entirely failed.* We have seen the detailed report of four of these cases, two of which died, and the other two seemed in no degree benefited by the treatment. A number of reputable physicians, not in government service, who were furnished with specimens of the bark, tried it with no better results.

Now comes Dr. Bliss, of Washington, onto the stage, and, *presto!* everything changes! He tries it in some six cases. In every one it works like a charm, all are immensely relieved, several are cured. The doctor hastens to herald to the world the glorious tidings that henceforth cancer shall be no more, for great is Cundurango and Bliss is its prophet!

We ask any reasonable person, is it likely that all these other observers were wrong and Dr. Bliss alone right; is it probable that they all reported falsely, and he only told the truth? What object could they have in making out a case against Cundurango? for, mind you, their experiments and reports were made before his, so that even jealousy of him could not have come in as a cause. Is there a reputable physician, nay, is there a decent man living who would not hail with joy the discovery of a cure for so dread a scourge of humanity as cancer? And yet some secular papers, amongst them even the *N. Y. Tribune*, echo Dr. Bliss' doleful plaint that the regular profession are "persecuting" him for his "discovery" of the virtues of this bark. Persecuting him because they report that they can find no efficiency in the drug, and because they have discovered that *four of Dr. Bliss' six cancer patients,* treated with Cundurango, *have died!!*

Was Dr. Bliss under any temptation to overestimate the value of the drug, to be misled himself and thus to mislead others? Judge for yourselves. Early in the history of this agitation, he dispatched his partner (whose name we believe is Keen) to South America to buy bark. While he was gone, Dr. Bliss vigorously "puffed" the article by circulars, newspaper articles, etc. The cargo, which is to make his fortune, having arrived, the doctor has abandoned his practice in Washington, and is to set up a "Laboratory" in New York. The *N. Y. Medical Record,* of September 15th, says: "A physician of this city who recently applied for four ounces [of Cundurango] was furnished with it, the bill being headed Bliss, Keen & Co., and the amount charged $50 00!!"

Let those who ask us be informed of these facts, and when "Bliss' Cancer Cure," put up in fancy bottles and covered with certificates of success, is "sold by all *respectable* druggists," some at least of the more intelligent in the community will know how to estimate its value.—*Kansas Med. Journal.*

*Cundurango.*—We have but little patience in chronicling of the progress of cundurango. We have already indicated our entire disbelief in all its claims. There is nothing upon which to build a show of faith. In the last number of the National Medical Journal, at Washington, we see that Dr. Bliss is expelled from the medical association, District of Columbia. We scarcely see how this could be otherwise. The whole thing is a fraud, sham, and swindle, and we deeply regret that any official influence should have been secured in its favor. The last quotation for cunderango is $100 per pound, only sold by Bliss, Keene & Co , New York.—*Cincinnati Lancet and Observer.*

*The Boston Medical and Surgical Journal,* of August 17th, has an article upon "Cundurango," by which it appears that stock in this new curative agent is already below par, and we are admonished that cancers will probably continue to afflict mankind for an indefinite period of time yet to come. Quacks will still flourish, and imposters still pocket enormous fees for curing simple tumors and for promising to cure malignant ones, and credulous people will still continue to be fleeced as of yore.—*Mo. Dental Journal.*

Cundurango has been consigned to an untimely grave. The quacks have adopted it. Dr. Bliss had best give it a long and last farewell, unless he has gone over to the ranks of charlatanism; for to praise it is charlatanism.—*Richmond Medical Journal.*

We published in the January number of the American Practitioner, a short account of this, the latest remedy, so-called, for cancer. Since then we have received several letters making inquiries concerning it. To one and all, we have to answer, that so far as we have been able to learn, cundurango is a complete failure.—*American Practitioner.*

Cundurango Wood is all the rage just now. We have foreborne to say anything about it, believing it was an intentional sensation, created for a special object. Dr. Bliss, of Washington, is at the top of the heap. His partner went to South America for a quantity of the wood. Meanwhile Dr. Bliss gets expectation on tip-toe by passing a gratuitous notice round the press that he will have plenty of it for sale at a certain date. Then like a very quack, he sends Mrs. Matthews, Schuyler Colfax, and lesser dignitaries, galloping round through the journals, proclaiming the virtues of the drug, to say nothing of the skill of Dr. Bliss. And several of our homœpathic journals bite the gudgeon with most shocking avidity. This is doubtless all right, but rumor has it hereabouts, not far from Dr. Bliss' early home, that somebody expects to scoop *sixty thousand dollars* on this enterprise. We shall see what we shall see.—*Ohio Medical and Surgical Reporter.*

